# Rapid Image Reconstruction of Structured Illumination Microscopy Directly in the Spatial Domain

**DOI:** 10.1109/JPHOT.2021.3053110

**Published:** 2021-01-20

**Authors:** Dan Dan, Zhaojun Wang, Xing Zhou, Ming Lei, Tianyu Zhao, Jia Qian, Xianghua Yu, Shaohui Yan, Junwei Min, Piero R. Bianco, Baoli Yao

**Affiliations:** 1 State Key Laboratory of Transient Optics and Photonics, Xi’an Institute of Optics and Precision Mechanics, Chinese Academy of Sciences, Xi’an 710119, China; 2 Xi’an Jiaotong University, Xi’an 710049, China; 3 Department of Pharmaceutical Sciences, College of Pharmacy, University of Nebraska, Medical Center, Omaha, NE 68198 USA

**Keywords:** Super-resolution microscopy, structured illumination, instant super-resolution imaging, spatial domain reconstruction

## Abstract

Super-resolution structured illumination microscopy (SIM) routinely performs image reconstruction in the frequency domain using an approach termed frequency-domain reconstruction (FDR). Due to multiple Fourier transforms between the spatial and frequency domains, SIM suffers from low reconstruction speed, constraining its applications in real-time, dynamic imaging. To overcome this limitation, we developed a new method for SIM image reconstruction, termed spatial domain reconstruction (SDR). SDR is intrinsically simpler than FDR, does not require Fourier transforms and the theory predicts it to be a rapid image reconstruction method. Results show that SDR reconstructs a super-resolution image 7-fold faster than FDR, producing images that are equal to either FDR or the widely-used FairSIM. We provide a proof-of-principle using mobile fluorescent beads to demonstrate the utility of SDR in imaging moving objects. Consequently, replacement of the FDR approach with SDR significantly enhances SIM as the desired method for live-cell, instant super-resolution imaging. This means that SDR-SIM is a “What You See Is What You Get” approach to super-resolution imaging.

## Graphical Abstract

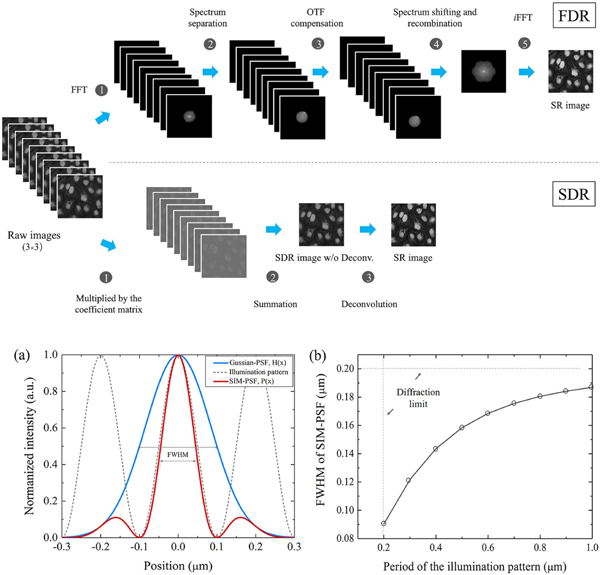

## 1. Introduction

In the conventional optical microscopy, using either wide-field or point-scanning illumination to detect scattering or fluorescence signals, the spatial resolution of the object’s image is confined by the well-known Abbe diffraction limit. This has been a barrier for optical imaging for over a century. In the past two decades, a variety of methods have been developed to overcome this limitation by exploiting the nature of light, adopting new imaging contrast mechanisms, or utilizing the nonlinear interaction effects between photons and molecules. The representative approaches for far-field super-resolution (SR) methods so far include stimulated emission depletion microscopy (STED) based on directly compressing the point spread function [1], [2]; single-molecule localization microscopy (such as PALM or STORM) by precisely localizing enormous point-source positions [3], [4], and special patterned illumination microscopy (such as structured illumination microscopy, SIM) via extending the detected spectrum in the spatial frequency domain or, called k-space based on the Fourier transform theory [5], [6].

As a wide-field (WF) microscopy technique, SIM features the merits of fast imaging speed, low excitation intensity, and a large field-of-view. Nowadays, SIM has been developed in various derivatives, such as three-dimensional [7]–[9], blind- [10], [11], Bayesian- [12], special-pattern [13], virtual-SIM [14], [15], and other variations [16], [17]. Moreover, some techniques based on the focal modulation scheme of laser scanning confocal microscopy (LSCM) are also regarded as a type of point-scanning SIM, such as the ISM [18], MSIM [19], ISIM [20], Rescan [21], and OPRA [22]. In this paper, SIM refers specifically to wide-field super-resolution microscopy with sinusoidal illumination patterns.

In such SIM systems, the basic workflow to generate a super-resolution image is built on the Fourier transform and spatial spectrum processing, which inevitably slows down the image reconstruction speed because of the complicated reconstruction process [23], [24]. Furthermore, improper treatments occurring in the frequency domain will cause unexpected artifacts in the recovered SR image [25]–[27]. Hence, one should pay careful attention to the frequency operation in the conventional, frequency-domain reconstruction (FDR) scheme [24].

To address the reconstruction speed and artifact problems, we introduce a new approach to super-resolution image reconstruction for SIM, named spatial domain reconstruction (SDR). The idea is inspired by the concept of a series expansion of a function in mathematics. That is, any function can be expressed by a linear superposition of a group of weighted complete orthogonal basis. In the SDR scheme, the SR image is attained by linear superposition of the patterned illuminated raw images with appropriately weighted coefficients that are derivable analytically. This concept appeared in the early works of Lucosz and So *et al.* [28], [29]. However, both of these studies were based on a theoretical model of structured illumination and were not rigorously tested experimentally.

In this paper, we present the processing routine of the SDR scheme to retrieve the SR image for SIM. The validity of the SDR approach is verified by both numerical simulation and its functionality demonstrated in different experiments. As there is no Fourier transformation and spectrum processing, the considerable burdens of the frequency operations like padding, filtering, and recombination in FDR are circumvented by the SDR method. Consequently, the simple data processing algorithm of SDR makes the image reconstruction speed significantly faster than that of FDR. As the proposed approach is fully compatible with conventional SIM systems, it can be applied to any linear SIM system, and conceivably can be further extended to non-linear SIM schemes to achieve theoretical, unlimited resolution as well.

## 2. Theory for SDR-SIM

In the linear SIM system, the image formed on the detector plane D(*r*) can be described as:

(1) $$D\left(r\right)=\int O\left(r^{\prime }\right)/\left(r^{\prime }\right)H\left(r-r^{\prime }\right)dr^{\prime },$$

where O(*r*), I(*r*), and H(*r*) represent the fluorescent emitter distribution of the object, the illumination intensity, and the point spread function (PSF) of the system, respectively. For simplicity without loss of generality, taking the one-dimensional case, in which the illumination field must be shifted several steps (indicated by the index *j*) towards one direction, (1) can be expressed in *x*-coordinate as followed:

(2) $$D_{j}\left(x\right)=\int O\left(x^{\prime }\right)/\left(x^{\prime }-\delta_{j}\right)H\left(x-x^{\prime }\right)dx^{\prime },$$

where *δ*_*j*_ is the shifting displacement in step *j*. The reconstructed SR image *R*_*SDR*(*x*)_ is expected to be attained by linear superposition of the designed phase-shifted patterned illumination images D_*j*_(*x*) multiplied with each corresponding coefficient function c_*j*_(*x*), that is,

(3) $$R_{SDR}\left(x\right)=\sum_{j=1}^{n}c_{j}\left(x\right)D_{j}\left(x\right)\;\;=\int O\left(x^{\prime }\right)\left[\sum_{j=1}^{n}c_{j}\left(x\right)/\left(x^{\prime }-\delta_{j}\right)H\left(x-x^{\prime }\right)\right]dx^{\prime }.$$

This formula can be regarded as a new image formation equation if the following equation is satisfied:

(4) $$\sum_{j=1}^{c}c_{j}\left(x\right)/\left(x^{\prime }-\delta_{j}\right)H\left(x-x^{\prime }\right)=T\left(x-x^{\prime }\right)H\left(x-x^{\prime }\right)=P\left(x-x^{\prime }\right),$$

where P(*x*) is regarded as a new PSF of the system. Since any periodic function can be expanded into a series of orthogonal complete basis, it is solvable to the expressions of c_*j*_(*x*) when an appropriate target function T(*x*−*x′*) is chosen. Here, we express I(*x*’-*δ*_*j*_) by the first order of orthogonal trigonometric basis (which means a sinusoidal fringe illumination), and assume the target function to be a normal cosine function, i.e.,

(5a) $$I\left(x^{\prime }-\delta_{j}\right)=I_{0}\left\{1+m\cos\left[2\pi k_{0}\left(x^{\prime }-\delta_{j}\right)+\varphi_{0}\right]\right\},$$

(5b) $$T\left(x-x^{\prime }\right)=1+\cos\left[2\pi k_{0}\left(x-x^{\prime }\right)\right],$$

Here I_0_, m, k_0_, and *φ*_0_ denote the mean intensity, modulation depth, spatial frequency, and initial phase of the illumination cosine fringe, respectively. In this circumstance, it can be proved that the least number of phase-shifting for the illumination fringe is three to solve out the coefficients c_*j*_(*x*), (i.e., *j* = 1, 2, 3), and further taking the three phase shifts to be 2*π*k_0_*δ*_*j*_ = {0, −2*π*/3, 2*π* /3}, we can obtain:

(6) $$\{\begin{array}{l} c_{1}\left(x\right)=\frac{1}{3l_{0}}\left[1+\frac{2}{m}\cos\left[2\pi k_{0}x+\varphi_{0}\right]\right]\\ c_{2}\left(x\right)=\frac{1}{3l_{0}}\left[1+\frac{2}{m}\cos\left(2\pi k_{0}x+\varphi_{0}+\frac{2\pi }{3}\right)\right]\\ c_{3}\left(x\right)=\frac{1}{3l_{0}}\left[1+\frac{2}{m}\cos\left(2\pi k_{0}x+\varphi_{0}-\frac{2\pi }{3}\right)\right] \end{array}.$$

Then, the reconstructed SR image and the new PSF can be attained:

(7a) $$R_{SDR}\left(x\right)=\frac{1}{3l_{0}}\sum_{j=1}^{3}\left\{1+\frac{2}{m}\cos\left[2\pi k_{0}x+\varphi_{0}+\frac{2\pi }{3}\left(j-1\right)\right]\right\}D_{j}\left(x\right),$$

(7b) $$P\left(x\right)=\left[1+\cos\left(2\pi k_{0}x\right)\right]\cdot H\left(x\right).$$

**Fig. 1.**
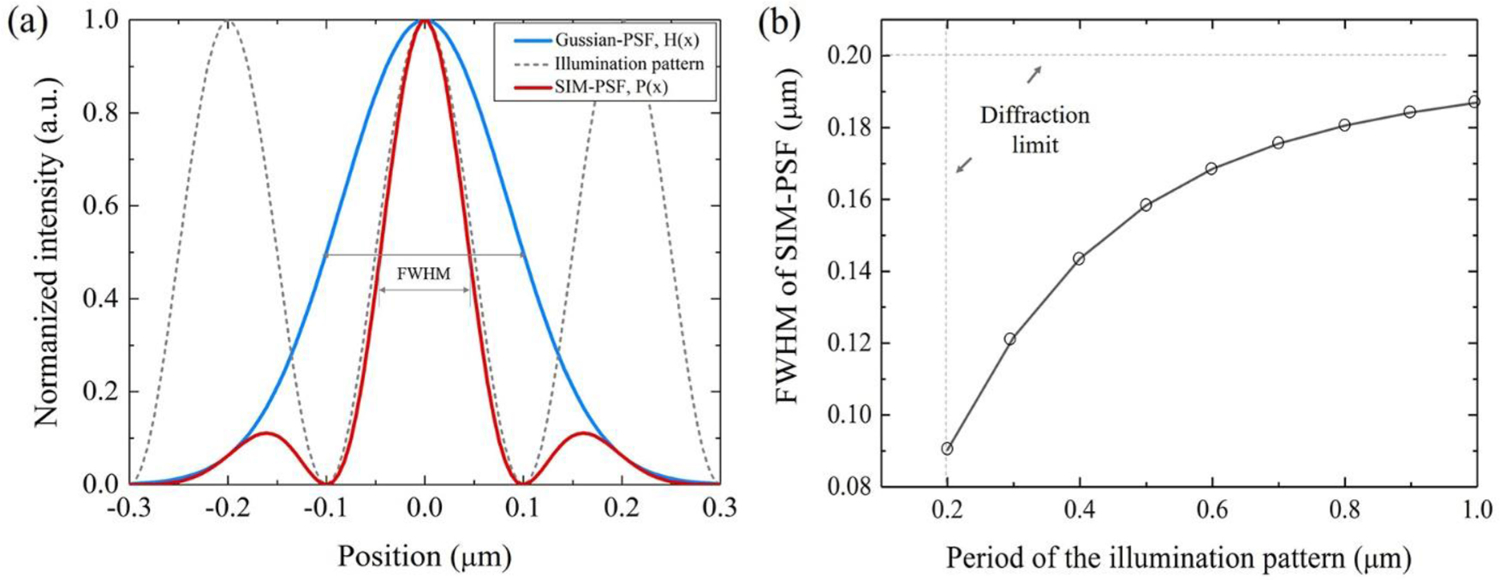
SIM attains a narrower PSF than conventional wide-field microscopy. (a) Schematic of PSF compression represented in the spatial domain. The PSF of a wide-field microscope is described by a normalized Gaussian function. (b) The variation of FWHM of SIM-PSF with the spatial frequency of the illumination pattern.

As indicated by (7b), the concept of the SDR approach is similar to PSF engineering, which improves the resolution by modulating and compressing the original PSF. As we know, the diffraction limit of a conventional light microscope is typically 200 nm. Assuming the PSF of a wide-field microscope is described by a Gaussian function, that is, $H\left(x\right)=e^{-x^{2}/\left(2\sigma^{2}\right)}$, where the full width at half maximum (FWHM) value of H(*x*) is equal to 200 nm. After modulating by a cosine function according to (7b), H(*x*) would be transformed into a new PSF, P(*x*), which is compressed narrower, as shown in Fig. 1(a). Here, the cosine function represents the distribution of structured illumination patterns. Its spatial frequency k_0_ is also diffraction-limited that it is not able to beyond the system cut-off frequency in value of 1/200 nm-1. The FWHM value of P(*x*) curve will decreases with the decreasing of the period of the illumination pattern T (T = 1/k_0_), as shown in Fig. 1(b). When T = 200 nm, i.e., the spatial frequency of the illumination pattern reaches the allowed maximum value, the FWHM value of P(*x*) approaches to its minimum value, approximately 90 nm here. When T = +∞, i.e., the case of uniform illumination, the FWHM value of P(*x*) curve goes to the diffraction limit value of 200 nm.

Theoretically, we can design any new PSF by selecting a suitable target function T(*x*) to modulate the original PSF, H(*x*). However, a more complex PSF generally requires a more complex structured illumination field I(*x*) and a greater number of raw images D_*j*_(*x*), because the illumination field needs to be expanded into more groups of orthogonal basis. The case of cosine illumination pattern and three equidistant steps phase-shifting is the simplest analytical solution.

## 3. Superiority of SDR-SIM

**Fig. 2.**
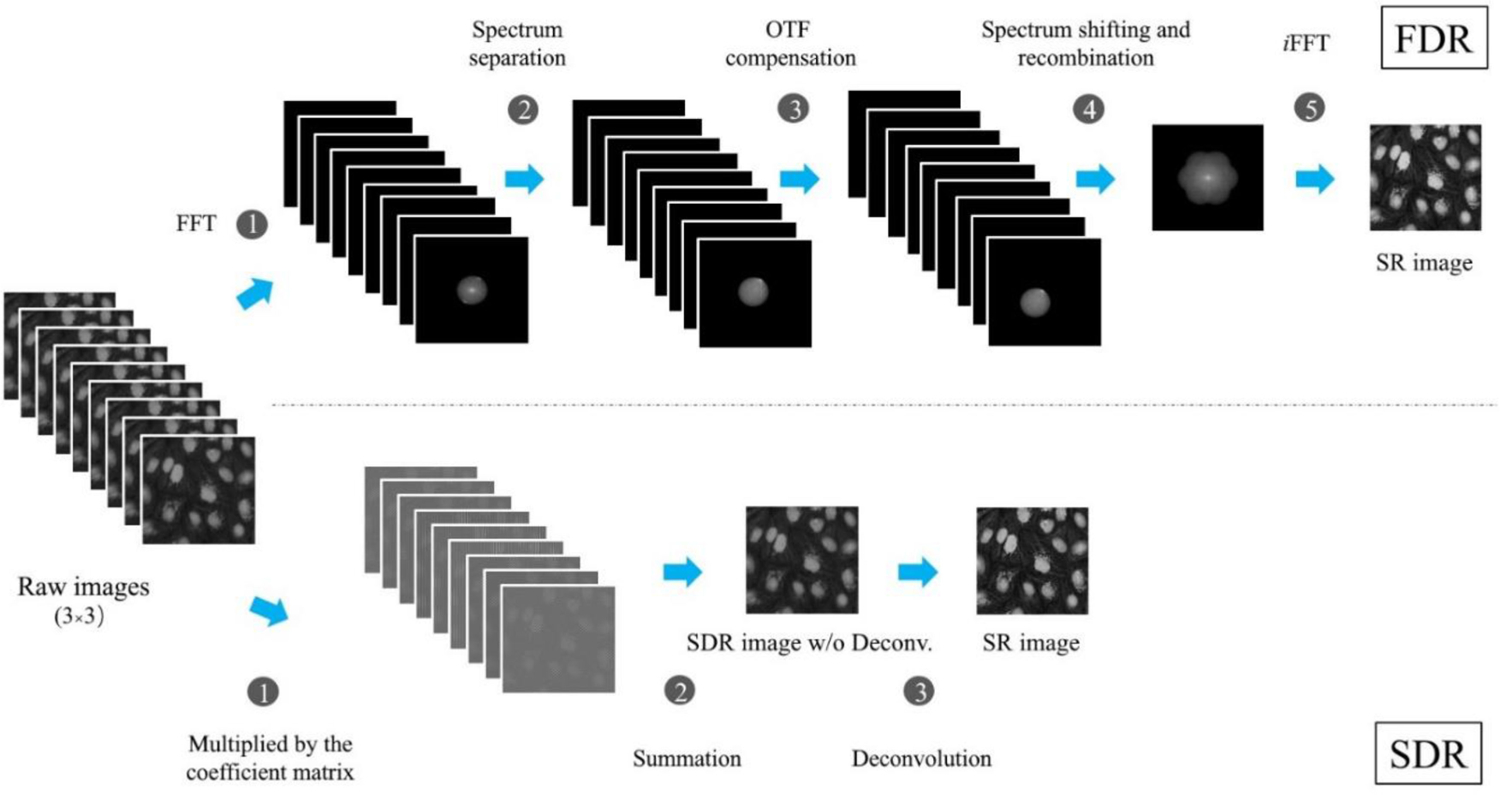
The SDR workflow is intrinsically simple compared to the complex, multi-step FDR approach. Top, the five steps of the FDR workflow to attain a super-resolution image. Bottom, the straightforward, 3-step SDR workflow.

The SR image reconstruction workflows for FDR- and SDR-SIM are illustrated in Fig. 2. In the FDR scheme, nine raw images from three orientation illumination patterns have to be Fourier transformed to obtain their spectra. All the spectra are then separated, OTF compensated, and recombined to form an enlarged isotropic spectrum, which corresponds to an SR image in the spatial domain after an inverse FFT (iFFT). The multiple operations of FFT and iFFT severely consume the processing time. In contrast, the SDR scheme is performed in the spatial domain with three simple operations: image multiplication, summation, and deconvolution. In addition, the multiplied coefficient matrixes are only dependent on the parameters of the structured illumination light field, and these can be pre-calculated to save the processing time. A comparison of the two pathways for image reconstruction suggests that due to its simplicity, SDR should be significantly faster than FDR in obtaining a super-resolution image.

**Fig. 3.**
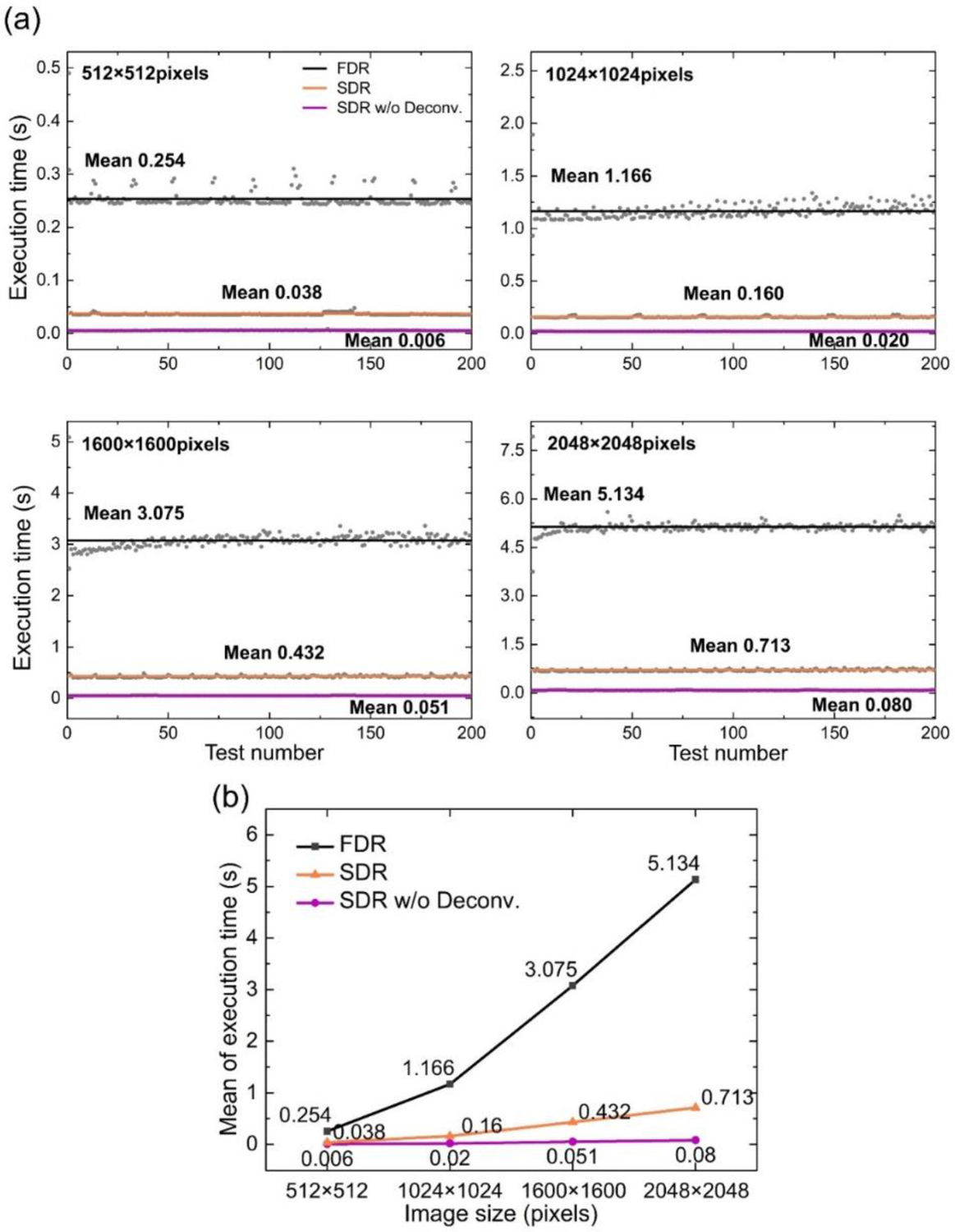
SDR produces SR images 7-fold faster than FDR. (a) The statistical execution time of 200 tests for different image sizes. (b) Variation of the mean execution time with the image size.

To test this, we compared the execution time for FDR, SDR, and SDR without deconvolution using four different image sizes of 512 × 512, 1024 × 1024, 1600 × 1600, and 2048 × 2048 pixels, respectively. Each image size was tested 200 times to determine the average execution time. For each SR image, there are nine structured illuminated raw images from three phase-shifts along three orientations as the input data. The expenditure time for pre-calculations such as the OTF transformed from PSF, the coefficient matrix of SDR, and the weighted factors for FDR spectra separation are excluded in the reconstruction time because they only depend on the system and the structured illumination parameters that are invariable during the reconstruction. The Wiener filtering-based deconvolution was used for both FDR and SDR. The reconstruction program is custom written with open-source C^++^ libraries of OpenCV and FFTW, running on a desktop computer (Intel i5–760 CPU, 16GB RAM, Windows 10 ×64). The comparison results are plotted in Fig. 3.

The results show that the mean value of execution time for SDR is 6.7∼7.2 fold faster than that of FDR for all four image sizes. The mean value of the execution time for SDR without deconvolution is about 42∼64 times faster than that of FDR. Further, the superiority of SDR is apparent when the image size becomes larger (Fig. 3b). The increase in the processing speed of SDR is due to the concise processing workflow.

## 4. Experimental Verification

**Fig. 4.**
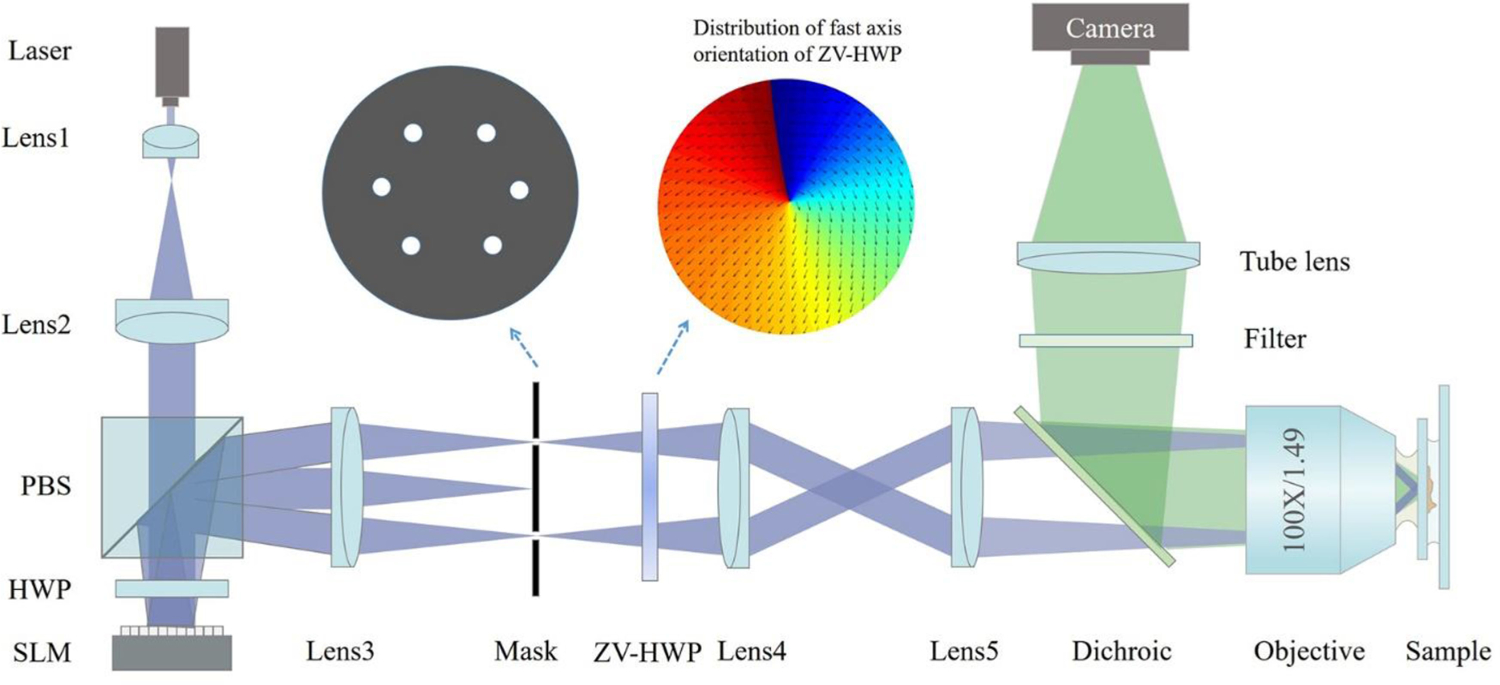
Schematic of the home-built SIM system. HWP: half-wave plate, ZV-HWP: zero-order vortex half-wave plate, SLM: spatial light modulator.

To demonstrate the effectiveness of SDR-SIM in producing super-resolution images at high-speed, the experiment was implemented in a home-built SIM system, illustrated in Fig. 4. The horizontally polarized laser beam with a wavelength of 561 nm (GEM561, Laser Quantum Inc., U.K.) is expanded and collimated by the Lens1 (*f* = 15 mm) and Lens2 (*f* = 200 mm) to illuminate the spatial light modulator (SLM) through a polarized beam splitter (PBS) and a half-wave plate (HWP). The ferroelectric liquid crystal SLM (QXGA-3DM, 2048 × 1536 pixels, 4kHz refresh rate, FDD Inc., U.K.) can quickly generate and shift different orientations diffractive gratings. The ±1st order diffraction beams are selected by a mask (hexagonal arranged pinholes array) for the generation of three orientation cosine fringes via interference. Here, a zero-order vortex half-wave plate (ZV-HWP, WPV10L, Thorlabs Inc., USA) is employed to simplify the complicated polarization control by a pair of wave retarder [30]. The beams relayed by a 4f system consisting of Lens4 (*f* = 175 mm) and Lens5 (*f* = 100 mm) are focused with a 100 × objective lens (Apo TIRF, NA1.49, Nikon Inc., Japan) to interfere on the sample to form the structured illumination. The fluorescence signal is reflected by a dichroic beamsplitter (Ex.561 nm/Em.610 nm) and filtered by a bandpass emission filter (580–630 nm) to be collected by a tube lens (*f* = 200 mm). The sCMOS camera with a maximum full-frame rate of 100 fps (Orca Flash4.0, 2048 × 2048 pixels, 16 bits gray level, Hamamatsu Inc., Japan) is used to capture the image. The sample is mounted on a motorized XY and piezo Z-axis translation stage (PZ-2150-XYLE-FT piezo Z system, ASI Inc., USA).

**Fig. 5.**
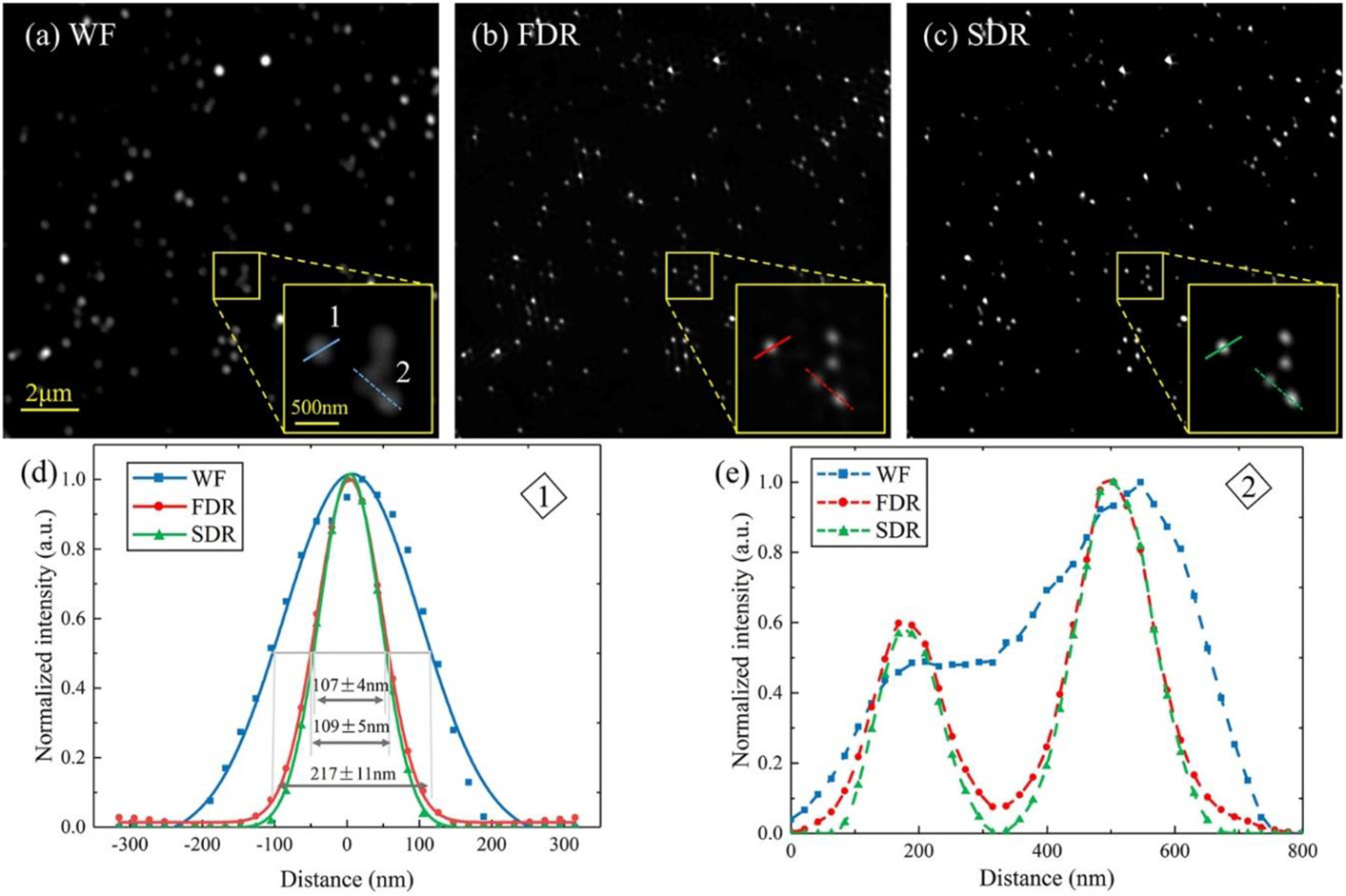
Resolution calibration of the system with 40 nm-diameter polystyrene fluorescence beads. (a)–(c) reconstructed images by wide-field, FDR- and SDR-SIM, respectively. (d)–(e) Intensity profiles along the marked lines in the magnified regions of each image.

First, we used 40 nm-diameter polystyrene fluorescence beads (Ex.565 nm/Em.580 nm, Thermo Fisher Scientific Inc., USA) as the sample to calibrate the spatial resolution of the system. Fig. 5 shows the measurement results for the wide-field, FDR- and SDR-SIM. To qualify the spatial resolution, we select 20 individual beads to plot their intensity profiles and fitted with Gaussian function to get the FWHMs, as shown in Fig. 5(d). The averaged FWHM values for wide-field, FDR- and SDR-SIM are 217±11 nm, 109±5 nm, and 107±4 nm, respectively. It can be seen that the resolution reconstructed by the FDR- and SDR-SIM are identical, and nearly double of the wide-field. We can also clearly see in the zoom-in region of the field in Fig. 5(a) that two beads are too closed to be resolved. But in the field of FDR- and SDR-SIM, they are separated distinctly. From Fig. 5(e), this can be presented by the intensity profiles.

**Fig. 6.**
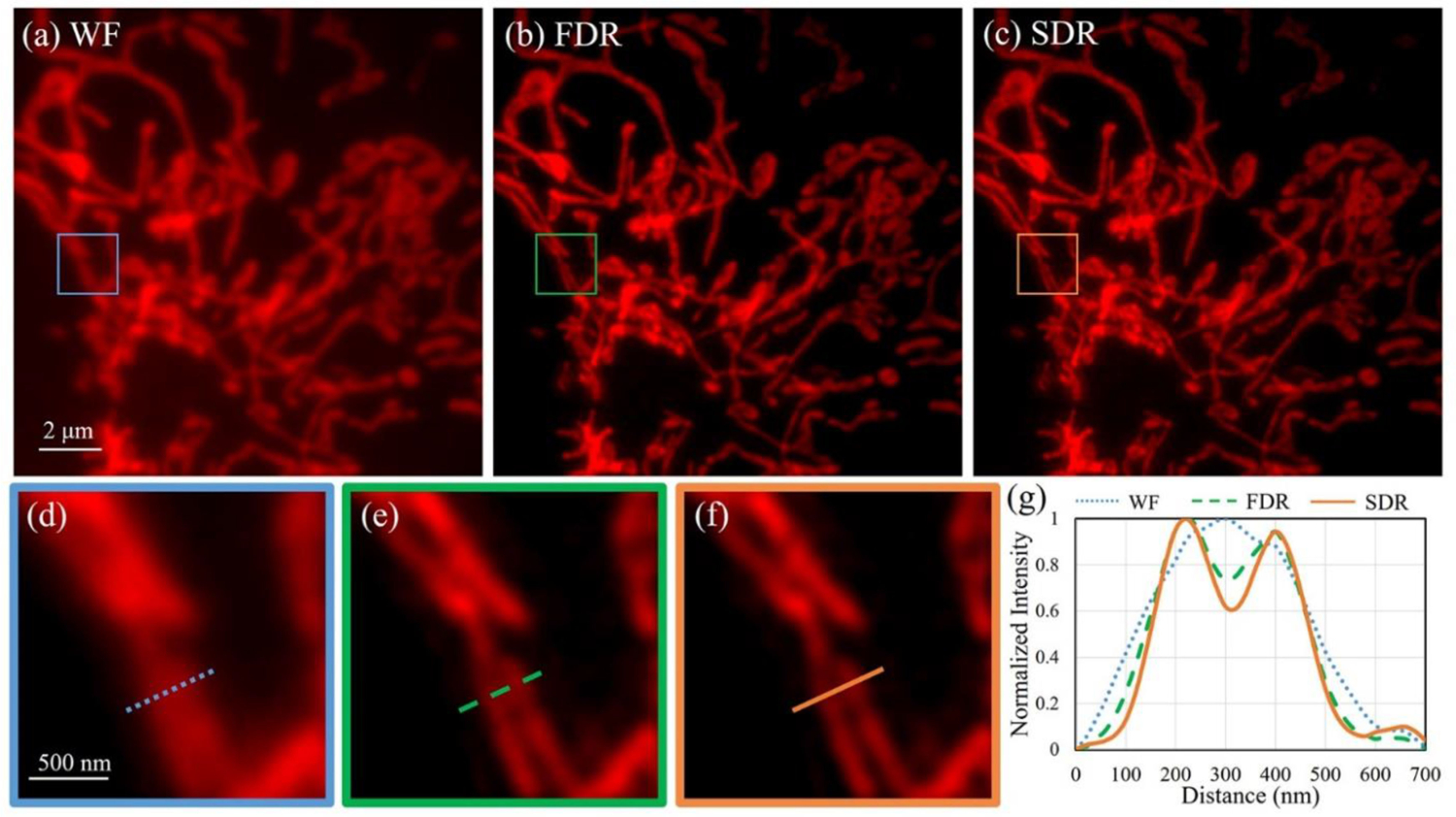
Imaging results of mitochondria in BPAE cell. (a)–(c) Images reconstructed by wide-field, FDR- and SDR-SIM, respectively. (d)–(f) The magnified views in the boxed regions in (a)–(c). (g) Intensity profiles along the marked lines in (d)–(f).

Next, a biological specimen was used to evaluate the performance of the system. The specimen is a slide of bovine pulmonary artery endothelial cells (BPAE, Thermo Fisher Scientific Inc., USA) with the mitochondria labeled by MitoTracker Red CMXRos (Ex.579 nm/Em.599 nm). The imaging results of wide-field, FDR- and SDR-SIM are presented in Fig. 6(a)–6(c), respectively. To make a clear comparison, we select a region of interest (ROI) in the field to zoom in, as shown in Fig. 6(d)–6(f). Two lines of adjacent mitochondria are discerned by the FDR- and SDR-SIM, whereas they are blurred in the wide-field image. This can also be distinguished in the plots of the intensity profiles along the marked lines, which shows that SDR is slightly better at discerning fine details than FDR (Fig. 6(g)).

To demonstrate the merit of SDR-SIM in imaging speed, we observe the Brownian motion of 100 nm fluorescence beads suspended in a mixture of water and glycerin at a ratio of 1:50:450. The mixture is pipetted onto a slide and covered by a 170*μ*m-thickness coverslip and sealed with nail polish. The Brownian motion of the beads is monitored and recorded by the SIM system in real-time. Each frame of the super-resolution image is reconstructed by computing nine raw images with the SDR method as described above. The raw image requisition speed is only limited by the frame rate and the sensitivity of the sCMOS camera because the refreshing rate of the fringes generation by the ferroelectric liquid crystal SLM is higher (4 kHz) than the camera.

The raw images were captured by the camera at speeds up to 100 fps at an image size of 2048 × 2048 pixels. The processing time of the SDR algorithm is 0.1s to output one frame of SR image from 9 raw images. Under these imaging conditions, the final maximum imaging speed (including data acquisition, processing, and display) of SDR-SIM is estimated to be 1/(0.01s × 9 + 0.1s) ≈ 5.2 fps. In practice, the longer exposure time of the camera owing to the weak signal will slow down the frame rate of the camera. In the demonstration of Brownian motion of 100 nm fluorescence beads, the exposure time of 3 ms and readout time of 10 ms of the camera is required to guarantee an adequate SNR of the image. In this case, the output SR video (see Visualization 1) for the dynamic beads is attained running at the frame rate of 4.6 fps, that is, 1/(0.013s × 9 + 0.1s). In sharp contrast, FDR-SIM is unable to reconstruct and display super-resolution frames instantly.

**Fig. 7.**
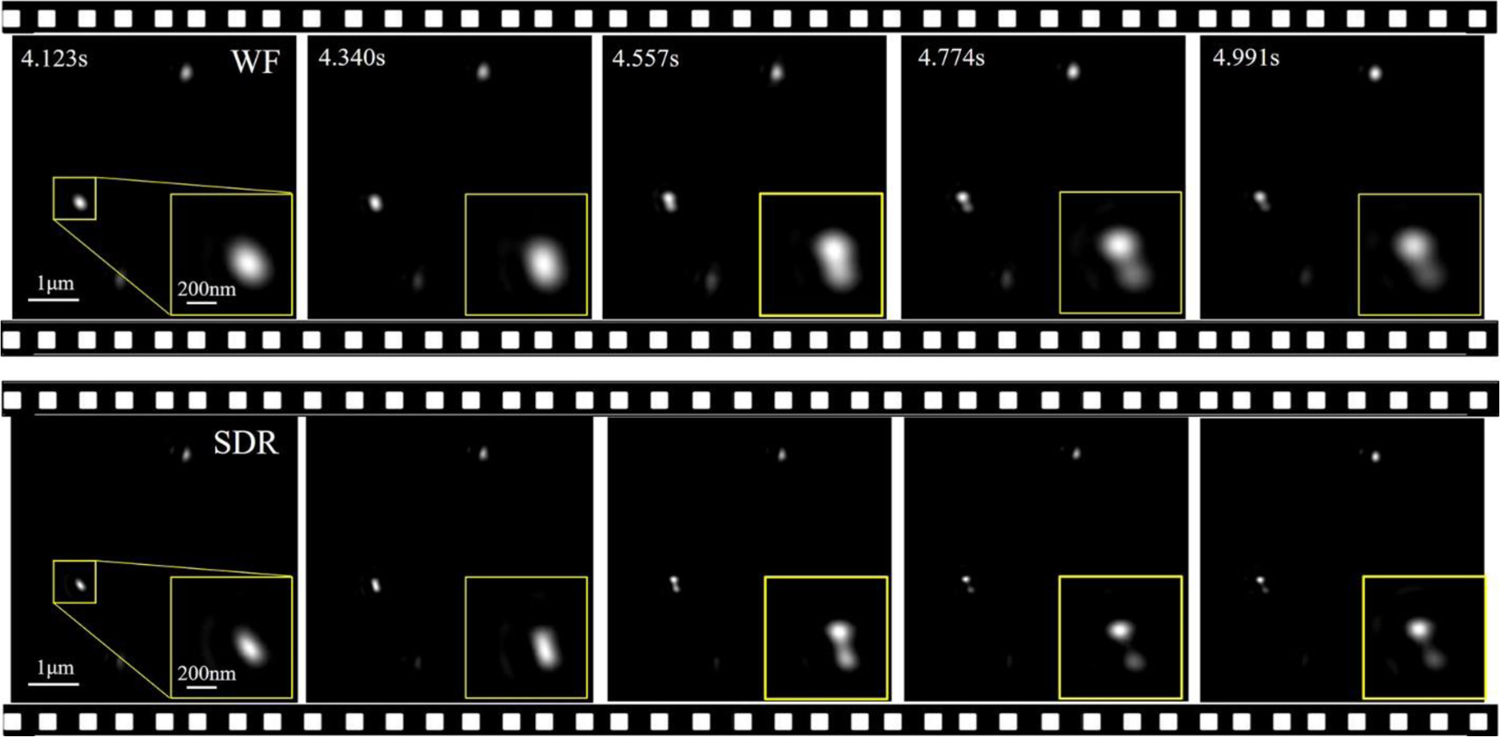
SDR-SIM enables resolution of 100 nm beads in the dynamic state. Five time-sequential frames in an interval of 217 ms are extracted from the SR video (see Supplementary Video). The zoom-in boxes display the relative position variance of two adjacent beads.

To show the merit of SDR-SIM against the wide-field microscope in spatial resolution in a dynamic scenario, we selected a region of interest in the full field of the video to track the motion behavior of two adjacent beads. Fig. 7 presents five time-sequential frames in an interval of 217 ms from the videos of the wide-field and SDR-SIM, respectively. As expected, the SDR-SIM produces clearer and sharper images of the beads compared to wide-field. Importantly, the separation process of the two adjacent beads (in the zoom-in boxes) from the overlapping to the separated state observed at t = 4.557s can only be shown by SDR-SIM.

We note that the SR video frame rate of SDR-SIM can be boosted by selecting the camera’s ROI working mode (only parts of pixels are exposed and readout) to reduce the image size and the field-of-view, depending on the observing requirement. Thus, at the 512 × 512 pixels working mode, the readout time of the camera is reduced to 2.5 ms. The SDR processing time can also be decreased to 16 ms. Then, the SR video-rate would be 1/(0.0055s × 9 + 0.016s) ≈ 15 fps.

## 5. Comparison of SDR-SIM With FairSIM

**Fig. 8.**
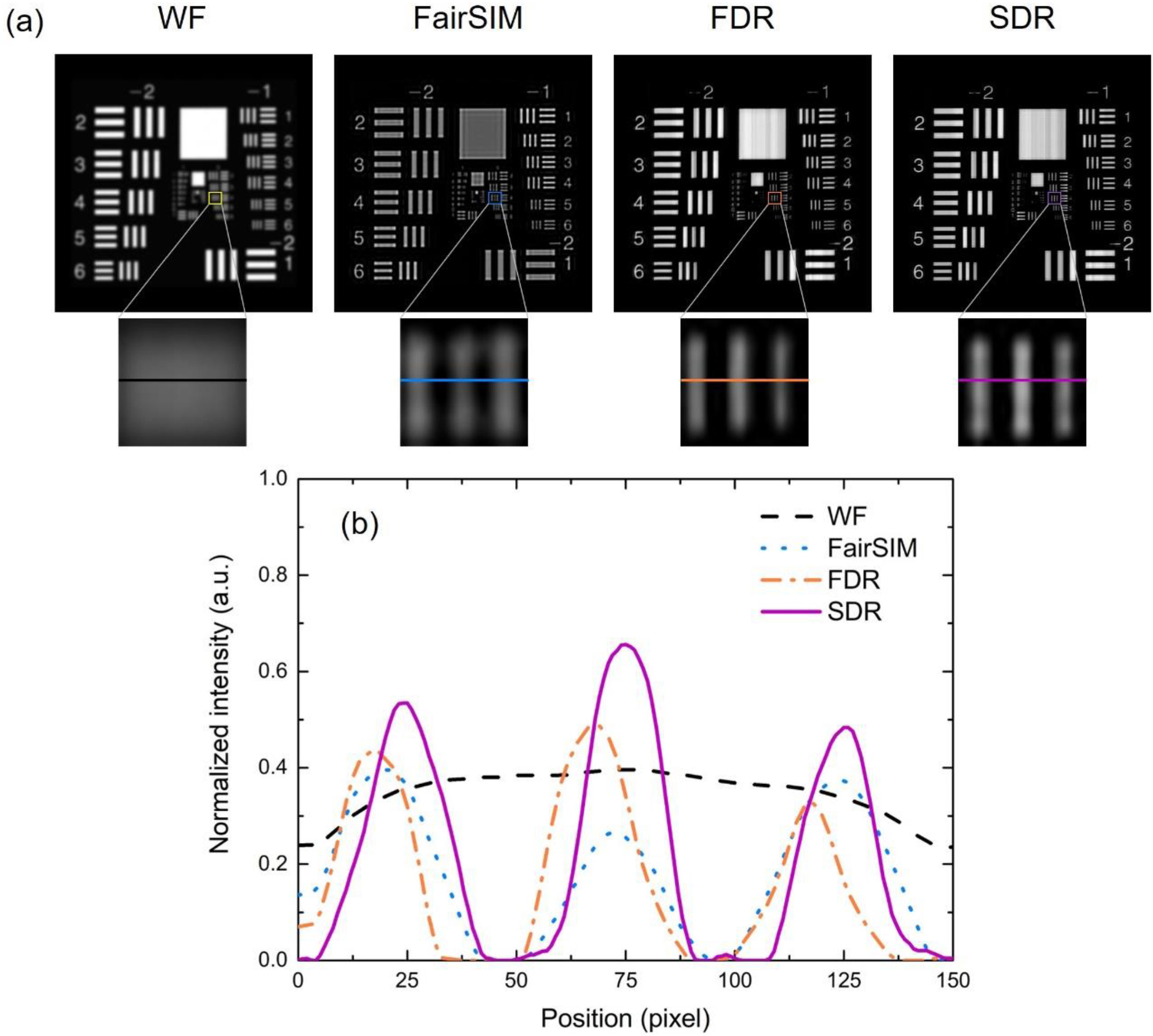
SDR-SIM produces the identical image as the widely-used FairSIM method. (a) Images with zoomed-in regions below. (b) Line profile plot of each zoom-in image in (a).

FairSIM, a broadly used program for reconstructing super-resolution images of SIM, is based on the principle of FDR [31]. To determine how SDR-SIM compares to FairSIM, we performed a comparison using a USAF resolution chart (Fig. 8). To be fair, we included FDR-SIM as well.

As the deconvolution process plays an important role in the resolution enhancement for structured illumination reconstructions, we employed the Wiener filtering-based deconvolution method in each approach. A comparison of the zoomed-in regions of each image in Fig. 8 demonstrates that SDR-SIM produces identical images to that produced by either FairSIM or FDR-SIM. This is further evident in the profile plot that the SDR-SIM can recover the same details as the FDR-SIM and FairSIM.

When the 2048 × 2048 image reconstruction times for each reconstruction method are compared, the superiority of SDR is realized. The reconstruction time for SDR is 0.7 s. In contrast, the reconstruction time FDR-SIM is 7-fold slower, taking 5.1s, and FairSIM is 23-fold slower taking 16s to produce the same image. Thus, SDR-SIM produces SR images significantly faster than currently available methods, and the image quality produced by SDR is identical to that of the widely-used FairSIM.

## 6. Conclusion

The primary conclusion of this study is that a novel structured illumination image reconstruction algorithm we call spatial domain reconstruction (SDR) produces super-resolution images of high quality and 7 to 23-fold more rapidly than the currently available methods. SDR achieves its goals using a far simpler approach to image reconstruction. The significantly enhanced image production speed and image quality produced by SDR is the result of two components of the scheme. First, image reconstruction is performed in the spatial domain thereby eliminating the need for Fourier transforms which slow the reconstruction process and can produce imaging artifacts. Second, the SDR SR image is attained by linear superposition of the patterned illuminated raw images with appropriately weighted coefficients that are derivable analytically. The method is elegant, simple, produces images rapidly, and is therefore ideally suited to live-cell imaging.

## Supplementary Material

supplemental material
